# Selective Whole-Blood Expression of Interferon-Stimulated Genes and TLR3/TLR7 in Type 1 Diabetes with Minimal Enterovirus-Targeted Amplification in Whole Blood

**DOI:** 10.3390/genes17091065

**Published:** 2026-09-02

**Authors:** Ilaria Galliano, Francesca Nardo, Davide Tinti, Cecilia Nobili, Paola Montanari, Cristina Calvi, Michela Trada, Luisa De Sanctis, Massimiliano Bergallo

**Affiliations:** 1 Department of Public Health and Pediatric Sciences, Medical School, University of Turin, Piazza Polonia 94, 10126 Turin, Italy; francesca.nardo@edu.unito.it (F.N.); paola.montanari@unito.it (P.M.); cristina.calvi@unito.it (C.C.); luisa.desanctis@unito.it (L.D.S.); massimiliano.bergallo@unito.it (M.B.); 2 Pediatric Laboratory, Department of Children’s Pathology and Care, Regina Margherita Children’s Hospital, Piazza Polonia 94, 10126 Turin, Italy; 3 Pediatric Diabetology Unit, City of Health and Science of Turin, Regina Margherita Children’s Hospital, Piazza Polonia 94, 10126 Turin, Italy; dtinti@cittadellasalute.to.it (D.T.); cecilia.nobili@unito.it (C.N.); mtrada@cittadellasalute.to.it (M.T.)

**Keywords:** type 1 diabetes, interferon signature, *TLR3*, *TLR7*, interferon-stimulated genes, innate immunity

## Abstract

Background: Enteroviruses (EVs) have been proposed as environmental triggers in type 1 diabetes (T1D), potentially through activation of innate immune pathways and type I interferon (IFN) responses. However, the relationship between detectable viral infection and IFN-related transcriptional responses remains unclear. Methods: In this cross-sectional, exploratory study, peripheral blood samples from individuals with new-onset T1D, established T1D, and healthy controls were analyzed by an enterovirus *5′NCR*-targeted RT-PCR assay and for the expression of innate immune receptors (*TLR3*, *TLR7*, *TLR8*), interferon-stimulated genes (*IFI27*, *IFIT1*, *ISG15*, *IFI44L*, *RSAD2*, *SIGLEC1*), and *FOXP3*, assessed as an exploratory marker related to regulatory T-cell biology potentially influenced by type I interferon signaling. Gene expression was assessed by quantitative RT-PCR, and comparisons were performed between groups. Results: Enterovirus-targeted amplification was observed in only one T1D sample under the applied assay conditions. In the setting of limited enterovirus-targeted amplification under the applied assay conditions, *TLR3* was significantly increased in established T1D, while *TLR7* was increased in both new-onset and established T1D, with the highest levels in established disease; these differences remained significant after correction for multiple comparisons across the full gene panel. *TLR8* was unchanged. Several of the selected interferon-stimulated genes showed group-specific expression patterns: *IFI27* and *RSAD2* were significantly increased in new-onset T1D, whereas *IFIT1* and *ISG15* were more evident in established T1D. *SIGLEC1* and *IFI44L* showed no significant differences. *FOXP3* transcript abundance was also increased in both diabetic groups, a finding that correlated with lymphocyte proportion and is interpreted with caution. Conclusions: These findings indicate group-specific differences in whole-blood transcript expression of selected RNA-sensing and IFN-related genes in T1D under conditions in which enterovirus-targeted amplification was observed in only one sample. Given the cross-sectional whole-blood design and the analytical limitations of the EV assay, these results are hypothesis-generating and do not establish the presence or absence of systemic enterovirus infection or functional pathway activation.

## 1. Introduction

Type 1 diabetes (T1D) is a chronic autoimmune disease characterized by the progressive destruction of insulin-producing pancreatic β-cells, leading to insulin deficiency and lifelong dependence on exogenous insulin therapy [1]. The pathogenesis of T1D is multifactorial, involving a complex interplay between genetic susceptibility and environmental triggers [1,2,3], as recently reviewed in detail [4]. Although genetic predisposition, particularly within the HLA region, accounts for a substantial portion of disease risk, epidemiological evidence such as incomplete concordance among monozygotic twins and rapidly increasing incidence rates highlights the importance of environmental factors [5], prompting large-scale prospective investigations such as the TEDDY study to systematically identify these triggers in genetically at-risk children [6].

Among environmental triggers, viral infections have long been implicated in the initiation and progression of β-cell autoimmunity [7,8]. Enteroviruses (EVs), particularly Coxsackie B viruses, have been consistently associated with T1D through epidemiological studies, detection of viral components in pancreatic tissue, and experimental models [9,10]. Viral capsid protein VP1 and enteroviral RNA have been detected in pancreatic islets of individuals with T1D, supporting the hypothesis of direct β-cell infection [8,11,12]. Pioneering evidence of direct pancreatic viral involvement came from the DiViD study, in which minimal tail resections were performed on six adult patients within a few weeks of T1D diagnosis. Enteroviral components were detected in the islets of all six individuals; however, the extent of infection appeared remarkably limited, with VP1-positive islets representing approximately 2% of the total examined, and the associated immune infiltrate being predominantly localized in the peri-insular region rather than within the islet core itself [8,13]. The marked predilection of enteroviruses for β-cells is likely not coincidental: these cells abundantly express the coxsackievirus and adenovirus receptor (CAR) [11], which constitutes a key entry point for coxsackievirus B serotypes. Furthermore, the well-documented inadequacy of β-cell innate immune signaling may render these cells not only more permissive to initial viral entry, but also less equipped to achieve complete viral clearance once infection is established [14]. Moreover, EVs are capable of establishing persistent infections characterized by low-level replication and limited cytopathic effects, which may allow long-term immune activation [15,16].

One of the major challenges in establishing a causal link between EV infection and T1D is the frequent lack of detectable viral RNA in peripheral blood [14]. Indeed, viral RNA degrades rapidly in plasma and fecal shedding typically persists for no longer than one to two weeks following acute infection in children, making the timing of sample collection a decisive factor in the reliability of these studies [14].

Evidence suggests that viral persistence may be restricted to pancreatic tissue or other compartments, resulting in systemic immune activation without measurable viremia [8,12,15]. Consistent with this concept, increased expression of type I IFN response markers in pancreatic islets has been associated with the presence of enteroviral capsid protein VP1 in donors with T1D, supporting a relationship between local antiviral responses and enteroviral markers at the tissue level [17]. This indicates that immune signatures related to viral sensing may persist even in the absence of detectable circulating virus. At the molecular level, persistence is facilitated by spontaneous 5′ terminal deletions in the viral genome that generate non-cytolytic, slowly replicating variants [14,15]. These deletion mutants tend to emerge preferentially in quiescent, non-dividing cells—a condition that characterizes pancreatic β-cells—and can coexist with minor wild-type viral populations that support their replication. In parallel, cell-to-cell spread via exosomes allows the virus to evade neutralizing antibodies without requiring cell lysis [14], thereby enabling sustained viral dissemination within the tissue over extended periods.

The innate immune system plays a central role in detecting viral infections through pattern recognition receptors (PRRs), including Toll-like receptors (TLRs) [18,19]. TLR3 recognizes double-stranded RNA (dsRNA), a replication intermediate of RNA viruses, whereas TLR7 and TLR8 detect single-stranded RNA (ssRNA) [20,21]. Upon activation, these receptors initiate signaling cascades involving adaptor molecules such as TRIF and MyD88, leading to activation of interferon regulatory factors (IRFs) and production of type I interferons (IFN-α/β) [18,19,22].

Type I interferons are key mediators of antiviral immunity and induce the expression of a large set of interferon-stimulated genes (ISGs) [22,23,24,25]. These genes encode proteins involved in viral restriction, RNA degradation, and modulation of immune responses. Among them, IFI27, IFIT1, and ISG15 are among the most rapidly induced and sensitive markers of interferon activation [26,27,28,29]. IFIT1 inhibits viral RNA translation by recognizing non-self RNA structures [29,30], ISG15 functions as a ubiquitin-like modifier involved in antiviral defense [31,32], and IFI27 has been associated with mitochondrial stress responses and apoptosis [33,34,35,36]. The six ISGs included in the present panel (IFI27, IFI44L, IFIT1, ISG15, RSAD2, and SIGLEC1) were selected a priori because this combination of transcripts has been previously used as a whole-blood transcriptional readout of type I interferon activity and for the calculation of type I IFN gene-expression scores [37,38]. The panel was therefore intended to provide a focused assessment of IFN-I-related transcription rather than a comprehensive representation of the entire ISG repertoire. Importantly, the six genes were analyzed individually in the present study, and only a subset showed differential expression across the study groups [37,38].

In T1D, an interferon transcriptional signature has been identified in peripheral blood and pancreatic islets, particularly at early stages of disease or prior to clinical onset [25,35]. Type I interferons contribute to disease pathogenesis by enhancing antigen presentation, increasing MHC class I expression on β-cells, and promoting immune cell recruitment [26,35]. Additionally, interferon signaling can induce β-cell stress and apoptosis, further contributing to disease progression [27,39,40].

However, heterogeneous ISG expression patterns have been reported in T1D, and individual ISGs may differ in induction kinetics, transcript stability, cellular source, and regulatory control [25,26,35]. Enteroviral proteins may also interfere with multiple components of type I IFN signaling: the EV proteases 2A and 3C can cleave signaling molecules including TRAF3, IRF7, TRIF, MAVS, and STAT1, while EV infection may upregulate SOCS3 and thereby attenuate downstream responses [14,16,41]. Nevertheless, these mechanisms cannot be inferred from the present data. Accordingly, the expression of the selected ISGs examined here should be interpreted as gene-specific transcriptional observations rather than as evidence of a globally restricted or selectively activated interferon pathway. Because type I interferon signaling can also influence regulatory T-cell stability and function, we additionally examined whether the innate immune and interferon-related transcriptional changes assessed here were accompanied by variation in *FOXP3* transcript abundance, used as an exploratory marker related to Treg biology. T1D is also associated with altered regulatory T-cell function, which is important for the maintenance of immune tolerance [42]. *FOXP3* is the principal transcription factor involved in Treg development and function [43]. However, *FOXP3* transcript expression does not necessarily reflect Treg abundance or suppressive activity, because it may also be transiently induced in activated conventional T cells [44], and inflammatory conditions can affect Treg stability and function [45]. We therefore assessed whole-blood *FOXP3* transcript abundance as an exploratory marker, without interpreting it as a direct measure of Treg number or suppressive capacity.

Based on these observations, the present study aimed to investigate the presence of enterovirus RNA in peripheral blood samples from T1D patients and to characterize the whole-blood transcript expression of selected innate immune receptors and interferon-stimulated genes. Particular emphasis was placed on determining whether the expression pattern of *TLR3*, *TLR7*, *IFI27*, *IFIT1*, and *ISG15* differed according to clinical disease status.

## 2. Materials and Methods

### 2.1. Study Population

The study population used to evaluate the selected targets was recruited at the Regina Margherita Children’s Hospital and was divided into two groups: patients with established diabetes and patients with new-onset diabetes. The diabetes group consisted of 48 patients, including 31 males and 17 females, with a median age of 11.84 years (IQR: 9.59–13.45 years). The new-onset diabetes group included 46 patients, comprising 19 males and 27 females, with a median age of 9.94 years (IQR: 5.95–12.54 years). New-onset type 1 diabetes patients (nT1D) were defined as subjects receiving a first clinical diagnosis of type 1 diabetes on the day of blood collection. Diagnosis was established according to ADA Standards of Care 2025 and ISPAD Clinical Practice Consensus Guidelines 2024, based on the presence of at least one of the following criteria: fasting plasma glucose ≥ 126 mg/dL after a minimum of 8 h of fasting or random plasma glucose ≥ 200 mg/dL accompanied by classic symptoms of hyperglycemia (polyuria, polydipsia, unintentional weight loss); or HbA1c ≥ 6.5%. All nT1D subjects tested positive for at least one diabetes-related autoantibody (GADA, IAA, IA-2A, or ZnT8A) and/or presented fasting C-peptide levels < 0.6 ng/mL. Diabetes-associated autoantibodies included GADA, IA-2A, IAA, ICA, and ZnT8A. Positivity was defined according to the laboratory-specific thresholds: GADA ≥ 5 U/mL, IA-2A ≥ 10 U/mL, ZnT8A ≥ 15 UA/mL, and categorical positive results for IAA and ICA. Among the 46 new-onset T1D patients, 40 (87.0%) were positive for at least one autoantibody, whereas 6 (13.0%) were negative for all available markers. Established type 1 diabetes patients (T1D) were defined as subjects with a confirmed T1D diagnosis according to the same criteria described above from at least one year prior to blood collection, on continuous insulin therapy, with at least one positive diabetes-related autoantibody documented at the time of original diagnosis; disease duration in this group was median 4.36 years (IQR 2.16–7.53), and HbA1c near the time of blood collection (last clinic visit) was median 6.90% (IQR 6.47–7.25), in addition to HbA1c at original diagnosis [46,47]. 

In parallel, 35 healthy controls (HC) were recruited for the IFN-I panel, and 25 healthy controls (HC) for the remaining targets of the project (*TLR3*, *TLR7*, *TLR8*, *FOXP3* and *GAPDH*), from the same hospital setting. These two HC cohorts were recruited in two sequential phases of the project, as the respective assay panels became available over time, and are therefore reported separately. All HC subjects were attending for elective purposes—including pre-surgical evaluation and diagnostic assessment to rule out genetic, metabolic, endocrinological, or nephrological conditions—and presented no laboratory abnormalities at the time of blood collection. For the IFN-I panel, the HC cohort comprised 21 males and 14 females, with a median age of 11.56 years (IQR 7.25–13.08); for the latter, the cohort comprised 13 males and 12 females, with a median age of 11 years (IQR 8.00–13.00). None had clinical or laboratory signs of active or recent infection. Individuals were not considered eligible for the HC group in the presence of any suspected or confirmed infectious disease, neoplastic condition, autoimmune disorder, neurological diagnosis, or pathological laboratory finding. Data on recent infections, vaccinations, and concomitant medications were not systematically collected for any of the study groups. Ethical approval was granted by the Institutional Review Board of the Azienda Ospedaliera-Universitaria Città della Salute e della Scienza, Turin (protocol code 0000274, approved 23 January 2025).

### 2.2. Sample Storage

For each participant, 200 μL of whole blood were mixed with 800 μL of RNApro stabilization solution (Biomole, Turin, Italy) [48] in a 1.5 mL Eppendorf tube and homogenized by vortexing. All samples were subsequently stored at −80 °C until further processing.

### 2.3. Total RNA Extraction

Total RNA was extracted from whole blood using the Maxwell automated extractor (Promega, Madison, WI, USA) with the RNA Blood Kit, which includes DNase treatment. RNA concentration and purity were assessed via UV spectrophotometry at 260/280 nm, using the Beer-Lambert law for quantification. NanoDrop (Thermo Fisher Scientific, Waltham, MA, USA) confirmed RNA concentrations within the acceptable range. Measurements were taken with 1 μL of RNA on an ND-1000 spectrophotometer at room temperature, with an A260/A280 ratio of 1.8–2.1 indicating high purity. RNA quality was assessed on the basis of RNA concentration, A260/A280 purity ratio, and successful amplification of the GAPDH reference transcript. RNA extracts were amplified without reverse transcription to rule out genomic DNA contamination and stored at −80 °C until use.

### 2.4. Reverse Transcription

Then, 400 ng of total RNA was reverse-transcribed with 2 µL of buffer 10×, 4.8 µL of MgCl2 (25 mM), 2 µL of Improm-II (Promega), 1 µL of RNase inhibitor 20 U/L, 0.4 µL of random hexamers (250 µM; Promega), 2 µL of mixed dNTPs (100 mM) (Promega), and dd-water in a final volume of 20 µL. The reaction mix was carried out in a GeneAmp PCR system 9700 Thermal Cycle (Applied Biosystems, Foster City, CA, USA) under the following conditions: 5 min at 25 °C, 60 min at 42 °C, and 15 min at 70 °C for the inactivation of the enzyme; the cDNAs were stored at −80 °C until use. All these procedures were performed according to the manufacturers’ protocols. Regarding control for genomic DNA contamination, we directly amplified RNA extract without reverse transcription.

### 2.5. Detection of Enterovirus RNA and Relative Quantification of TLR3, TLR7, TLR8, and FOXP3 

Enterovirus RNA detection was performed by real-time RT-PCR targeting the conserved 5′ non-coding region (5′NCR) of the enterovirus genome. Total RNA extracted from whole blood was reverse-transcribed as described above, and cDNA was amplified using specific primers and probe for the enteroviral 5′NCR region. Amplification reactions were carried out using the ABI PRISM 7500 real-time system (Life Technologies, Austin, TX, USA). Each run included a no-template control to monitor for contamination, and no amplification was observed in any no-template control. All clinical samples were analyzed in triplicate. Enterovirus RNA was classified as detected when a reproducible amplification curve was observed in all three technical replicates. Samples showing no amplification in any of the three replicates were classified as having no enterovirus-targeted amplification under the applied assay conditions. One sample showed reproducible amplification in all three technical replicates, with Ct values ranging from 27 to 28, whereas no amplification curve was observed for the remaining samples. The *5′NCR* assay was used exclusively to assess enterovirus-targeted amplification in whole-blood-derived RNA and was not normalized to *GAPDH*. An external positive control was not included, and a formal analytical limit of detection and amplification efficiency were not established in the present study. In parallel, relative quantification of *TLR3*, *TLR7*, *TLR8* and *FOXP3* transcripts was achieved by real-time TaqMan PCR amplification and normalization to glyceraldehyde-3-phosphate dehydrogenase (*GAPDH*), which was chosen as a reference gene, using the ABI PRISM 7500 real-time system (Life Technologies, Austin, TX, USA). Then, 40 ng of cDNA was amplified in a 20 µL total volume reaction containing Go-Taq mastermix probe (Promega). For all targets, reactions included 500 nM of specific primers and 200 nM of specific probes, whereas FOXP3 amplification was performed using 900 nM of specific primers and 250 nM of specific probes. The primers and probes used for enterovirus RNA detection and gene expression analysis are reported in Table 1.

The assays used probes and primers designed by Primer Express TM software version 3.0 (Applied Biosystems, Foster City, CA, USA). Basic Local Alignment Search Tool (BLAST, Version 2.12.0) analysis confirmed no cross-reaction. Amplifications were run on a 96-well plate at 95 °C for 2 min, followed by 45 cycles at 95 °C for 15 s and at 60 °C for 1 min. All gene-expression assays were performed in triplicate. Mean Ct values were calculated from valid technical replicates; a replicate was considered anomalous and excluded when its Ct value fell outside the mean Ct of the triplicate ± 2 standard deviations. Clinical samples and healthy controls were amplified within the same runs to minimize inter-run variability, and all analyses were performed by qualified personnel trained in molecular biology procedures. Target-specific standard curves were not generated; therefore, experimental amplification efficiencies were not independently determined. Relative target-gene expression was calculated using the 2^−ΔΔCt method, with the mean ΔCt of the healthy-control group used as the calibrator. 

### 2.6. Transcription Assessment of Selected Type I Interferon-Related Transcripts by RT-qPCR

GAPDH was chosen as the reference gene due to its stability and previous use in our studies [49,50,51]. Relative quantification of the selected IFN-I-related transcripts was performed using the ABI PRISM 7500 real-time system (Thermo Fisher Scientific).

Fifty ng of RNA were reverse transcribed and amplified in a single one-step RT-qPCR reaction using the IFN-I mRNA expression kit (BM-023, BioMole) in a 20 μL reaction, as opposed to the two-step protocol (separate reverse transcription followed by amplification of stored cDNA) described in Section 2.4 and Section 2.5 for the *TLR3*/*TLR7*/*TLR8*/*FOXP3*/enterovirus panel. The BM-023 kit evaluated six IFN-I ISGs (*IFI44L*, *ISG15*, *IFIT1*, *RSAD2*, *SIGLEC1*, *IFI27*). 

Reactions were performed in a 96-well plate with the following thermal profile: 50 °C for 10 min, 95 °C for 10 min, followed by 40 cycles of 95 °C for 10 s and 60 °C for 30 s. Each sample was run in triplicate. Relative quantification was done using the 2^−ΔΔCt method [52], with the mean ΔCt of the healthy-control group used as the calibrator, and results were expressed as relative quantification (RQ) in arbitrary units.

The six interferon-stimulated genes included in the panel were analyzed individually in order to characterize gene-specific patterns of interferon-related transcript expression across the study groups.

All analyses were conducted in a Biosafety Level 2 (BSL-2) laboratory, following NIH and WHO guidelines [53,54].

### 2.7. Statistical Analysis

Data distribution was assessed using the Shapiro–Wilk test. As all targets mRNA expression values did not follow a normal distribution, non-parametric statistical methods were applied. Raw GAPDH Ct values were compared among the study groups using the Kruskal–Wallis test to assess the relative stability of the reference transcript. Comparisons among diabetic patients, patients with new-onset diabetes, and healthy controls were performed using the Kruskal–Wallis test, followed by Dunn’s multiple comparisons test. To account for multiplicity across the complete panel of ten targets (three TLRs, six ISGs, and FOXP3; 30 pairwise comparisons in total), Benjamini-Hochberg false discovery rate (FDR) correction was applied to the complete set of exact Dunn’s multiple-comparison *p*-values. Post-hoc Spearman correlation analyses were performed between target transcript expression and leukocyte differential counts in both diabetic cohorts and, for established T1D, lymphocyte subpopulations assessed by flow cytometry. Full results are reported in Supplementary Table S3. Age and sex distributions between the new-onset and established T1D groups were compared using the Mann-Whitney U test and chi-square test, respectively. Given the exploratory design and the available sample size, no additional post-hoc clinical subgroup stratification was performed in the absence of a prespecified biological hypothesis, to avoid unstable estimates and further inflation of multiple testing. A *p*-value < 0.05 (nominal, or FDR-adjusted where indicated) was considered statistically significant. All statistical analyses were performed using GraphPad Prism software (Version 7, GraphPad Software, La Jolla, CA, USA), with FDR correction and correlation analyses performed in Python, Version 0.14.5 (SciPy/statsmodels).

## 3. Results

### 3.1. Study Population

The main clinical characteristics of T1D and nT1D subjects enrolled in this study are summarized in Table 2.

The number of evaluable samples varied slightly among individual molecular targets because of inadequate RNA yield, amplification failure, or target-specific missing data. Exact evaluable sample sizes for each target and cohort are reported in Supplementary Table S1.

### 3.2. Enterovirus RNA Detection

Enterovirus RNA was investigated in whole blood samples by real-time RT-PCR targeting the conserved 5′NCR region of the enterovirus genome. Enterovirus-targeted amplification was observed in one sample from the established T1D cohort, with concordant Ct values of 27–28 across all three technical replicates. No amplification was observed under the applied assay conditions in the remaining established T1D, new-onset T1D, or healthy-control samples.

### 3.3. TLR3, TLR7, and TLR8 mRNA Expression

Raw *GAPDH* Ct values did not differ significantly among the study groups (Kruskal–Wallis test, *p* = 0.1128), supporting the relative stability of *GAPDH* under the present experimental conditions.

To evaluate the transcriptional status of innate immune receptors implicated in type 1 diabetes, we quantified mRNA expression levels of *TLR3*, *TLR7*, and *TLR8* in all three groups. Median expression values (IQR 25–75%) were as follows: *TLR3*: 1.2 (0.73–1.8), 1.6 (1.09–2.49), and 2.8 (2.21–4.55) for HC, nT1D and T1D respectively; *TLR7*: 1.0 (0.90–1.16), 1.4 (1.10–2.17), and 2.4 (1.68–2.94) for HC, nT1D and T1D respectively; *TLR8*: 1.0 (0.76–1.23), 1.2 (0.92–1.56), and 1.1 (0.81–1.6) for HC, nT1D, and T1D patients, respectively.

**Figure 1:**
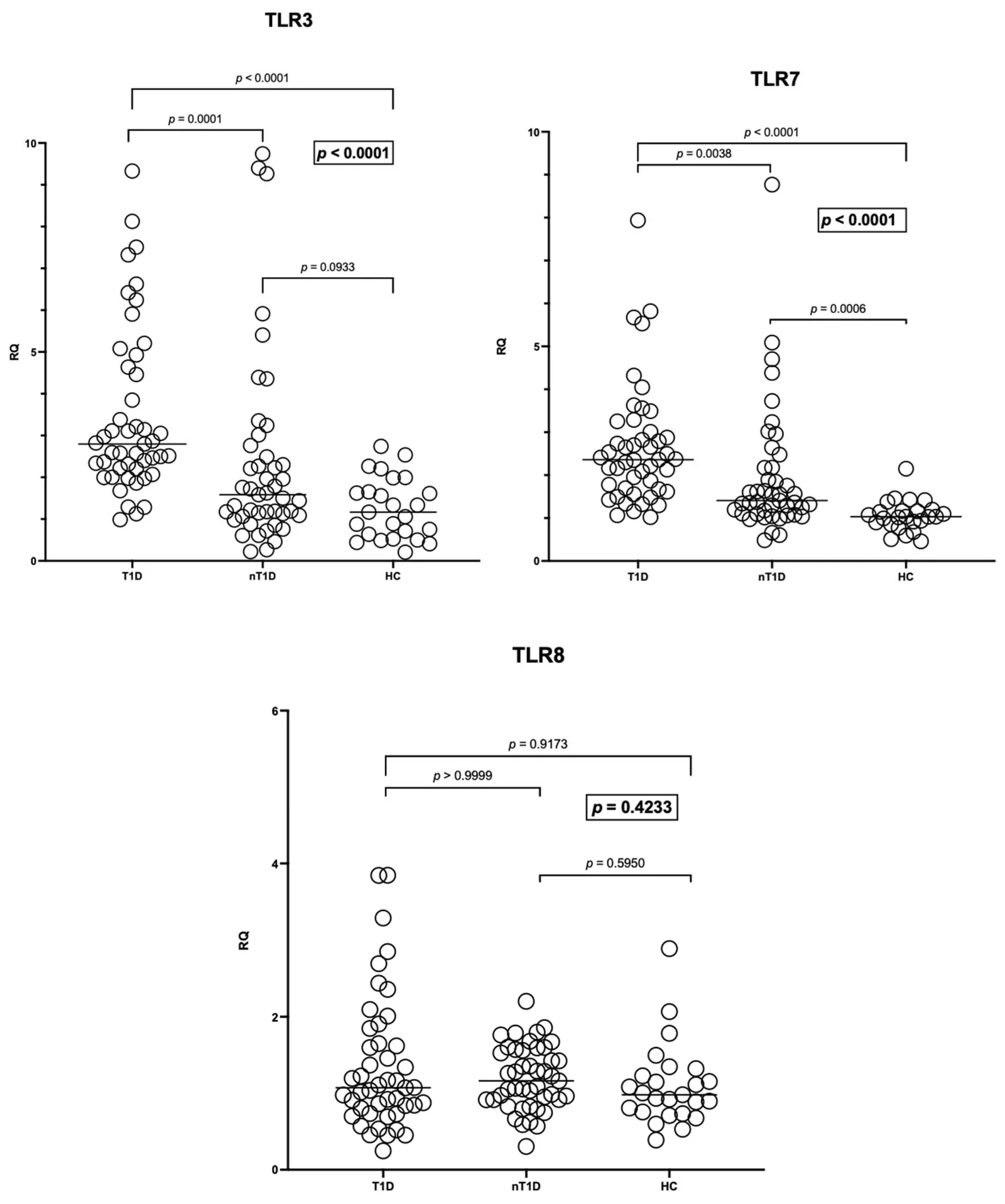
Different mRNA expression levels of *TLR3*, *TLR7* and *TLR8* in healthy controls (HCs), type 1 diabetes (T1D) and new-onset type 1 diabetes (nT1D) patients. Analysis of *TLR3*, *TLR7* and *TLR8* expression was normalized to *GAPDH* expression. Data are presented as RQ (relative quantification) calculated using the ∆∆Ct method. Horizontal lines represent median values. Statistical analysis: Kruskal–Wallis test (boxed *p* values), followed by Dunn’s multiple comparisons test; all significant comparisons remained significant after Benjamini-Hochberg FDR correction across the full 30-comparison gene panel (Supplementary Table S2).

*TLR3* expression was significantly elevated in T1D compared with HC (*p* < 0.0001) and nT1D (*p* = 0.0001), while no significant difference was observed between nT1D and HC (*p* = 0.0933). *TLR7* showed a similar upregulation pattern, with significant differences across all pairwise comparisons: T1D vs. HC (*p* < 0.0001), T1D vs. nT1D (*p* = 0.0038), and nT1D vs. HC (*p* = 0.0006). In contrast, TLR8 did not differ significantly across any pairwise comparison (T1D vs. nT1D, *p* > 0.9999; T1D vs. HC, *p* = 0.9173; nT1D vs. HC, *p* = 0.5950), as confirmed by the Kruskal–Wallis test (*p* = 0.4233). Taken together, TLR3 and TLR7 expression was higher in established T1D and, for TLR7, also in new-onset T1D, compared with HC, whereas TLR8 expression was comparable across all three groups (Figure 1). All significant *TLR3* and *TLR7* comparisons remained significant after FDR correction across the full gene panel (Supplementary Table S2).

### 3.4. FOXP3 Expression Levels

**Figure 2:**
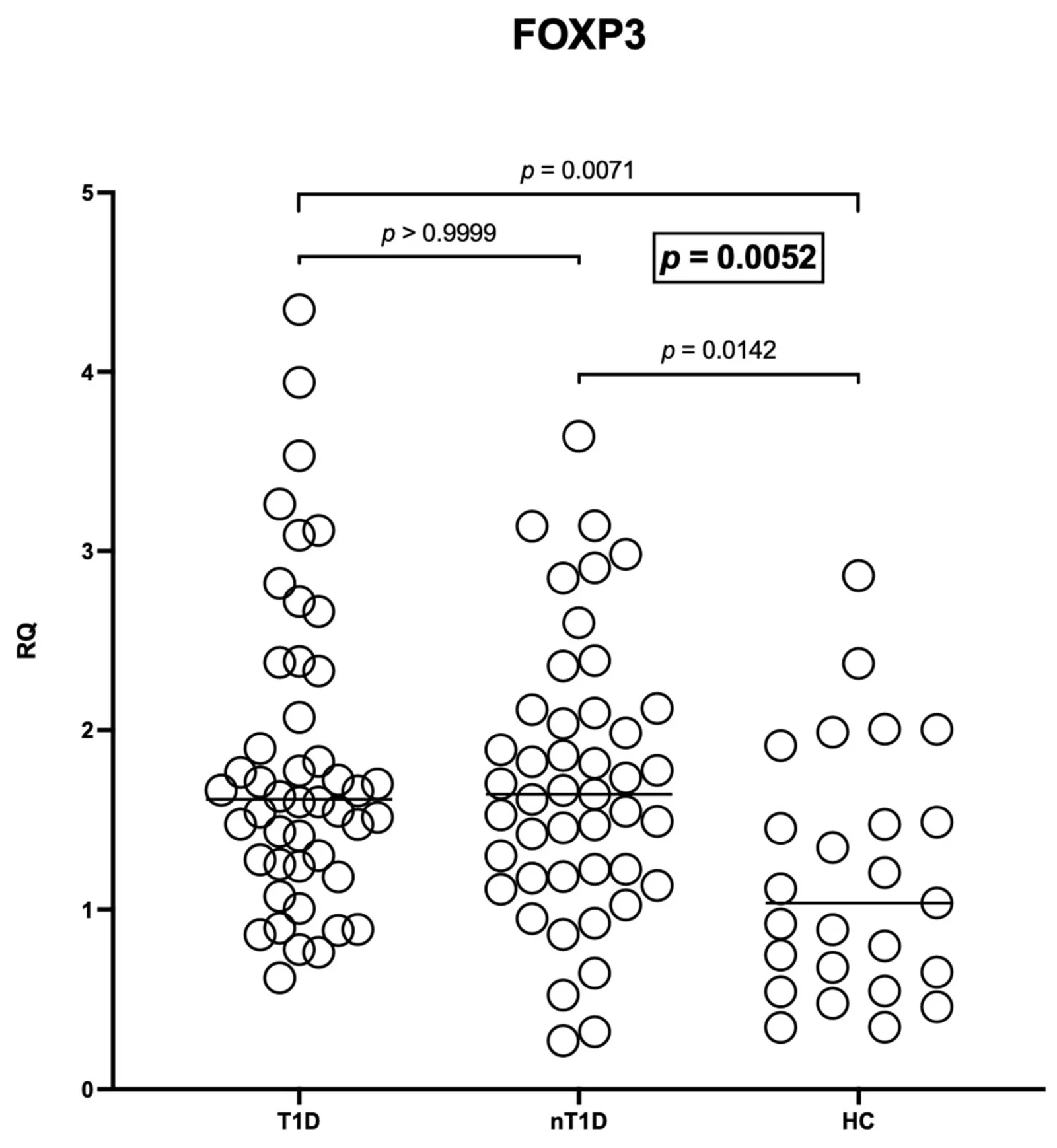
mRNA expression levels of *FOXP3* in healthy controls (HCs), type 1 diabetes (T1D) and new-onset type 1 diabetes (nT1D) patients. *FOXP3* expression was normalized to *GAPDH* expression. Data are presented as RQ (relative quantification) calculated using the ∆∆Ct method. Horizontal lines represent median values. Statistical analysis: Kruskal–Wallis test (boxed *p* values), followed by Dunn’s multiple comparisons test; all significant comparisons remained significant after Benjamini-Hochberg FDR correction across the full 30-comparison gene panel (Supplementary Table S2).

We subsequently analyzed *FOXP3*, the master regulator of regulatory T cells (Tregs). Median expression (IQR 25–75%) was 1.04 (0.54–1.33), 1.61 (1.26–2.26), and 1.64 (1.18–2.10) for HC, T1D, and nT1D patients, respectively. Expression was significantly elevated in both T1D (*p* = 0.0071) and nT1D (*p* = 0.0142) compared with HC, while no significant difference was observed between the two diabetic groups (*p* > 0.9999). Thus, in contrast to the group-specific pattern observed for *TLR3* and *TLR7*, *FOXP3* expression was similarly elevated in both diabetic groups (Figure 2). Its associations with leukocyte composition are reported in Section 3.6 and Supplementary Table S3.

### 3.5. Expression of Selected Type I Interferon-Stimulated Genes

To evaluate the transcriptional profile of type I interferon-stimulated genes, mRNA expression levels of *IFIT1*, *IFI27*, *RSAD2*, *IFI44L*, *ISG15*, and *SIGLEC1* were quantified in whole blood across all three groups. Median expression values (IQR 25–75%) were as follows—*IFIT1*: 1.06 (0.76–1.38), 1.05 (0.43–1.64), and 1.49 (1.05–2.17) for HC, nT1D, and T1D patients, respectively; *IFI27*: 0.82 (0.39–1.44), 2.50 (1.10–5.89), and 0.97 (0.55–2.30) for HC, nT1D, and T1D patients, respectively; *RSAD2*: 0.98 (0.69–1.52), 2.38 (1.42–4.23), and 0.95 (0.55–1.69) for HC, nT1D, and T1D patients, respectively; *IFI44L*: 1.02 (0.73–1.41), 1.33 (0.87–5.89), and 0.91 (0.60–1.69) for HC, nT1D, and T1D patients, respectively; *ISG15*: 0.97 (0.72–1.36), 1.20 (0.79–1.70), and 1.37 (0.92–2.42) for HC, nT1D, and T1D patients, respectively; *SIGLEC1*: 1.03 (0.69–1.35), 1.25 (0.81–2.08), and 1.15 (0.66–2.00) for HC, nT1D, and T1D patients, respectively.

**Figure 3:**
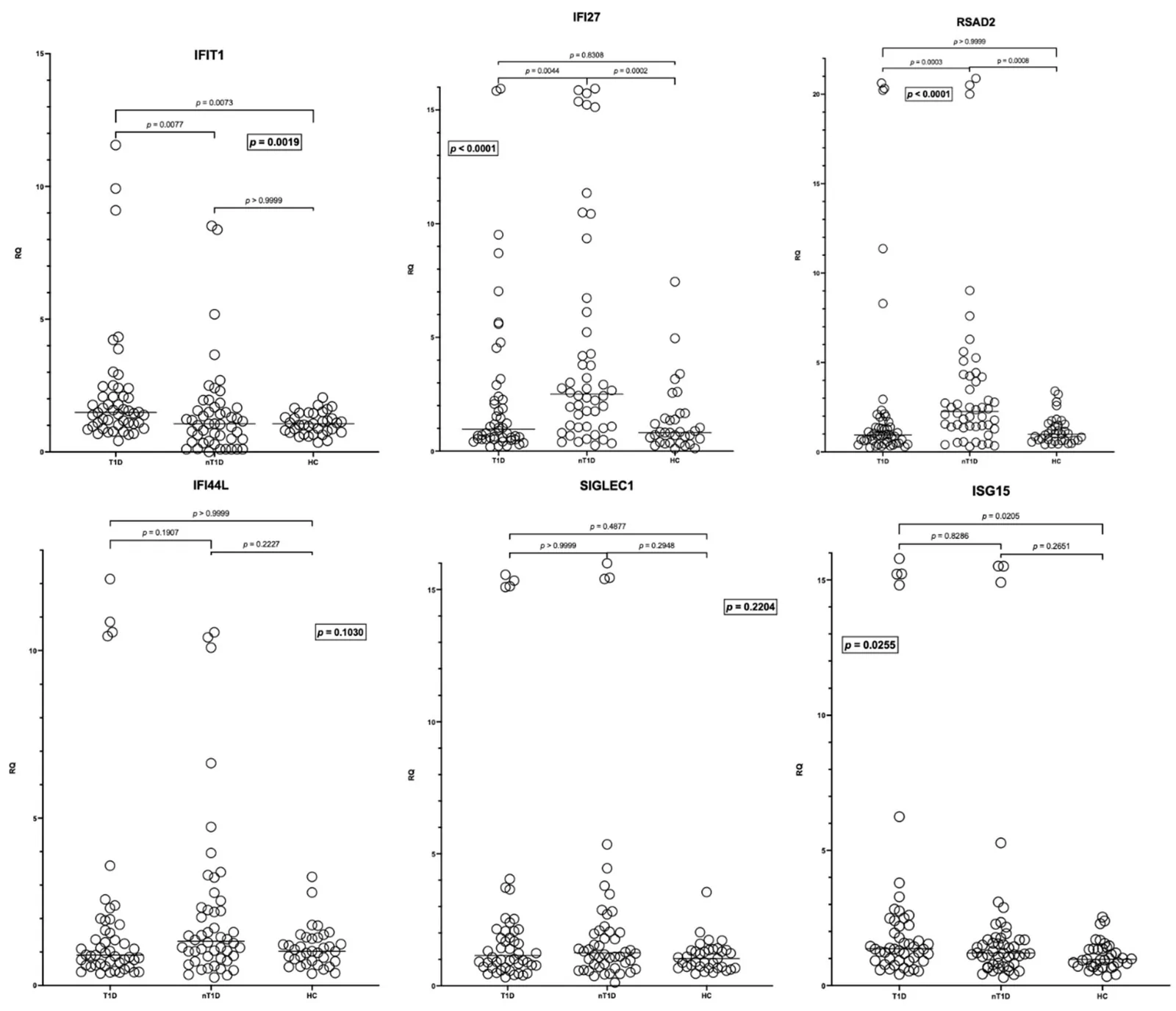
mRNA expression level of *IFIT1*, *IFI27*, *RSAD2*, *IFI44L*, *SIGLEC1* and *ISG15* in healthy controls (HCs), type 1 diabetes (T1D) and new-onset type 1 diabetes (nT1D) patients. Analysis of all targets expression was normalized to *GAPDH* expression. Data are presented as RQ (relative quantification) calculated with the ∆∆Ct method. Horizontal lines represent the median values. Statistical analysis: Kruskal–Wallis test (boxed *p* values), followed by Dunn’s multiple comparisons test; all significant comparisons remained significant after Benjamini-Hochberg FDR correction across the full 30-comparison gene panel (Supplementary Table S2).

*IFIT1* was significantly upregulated in T1D relative to both nT1D (*p* = 0.0077) and HC (*p* = 0.0073), with no difference between the latter two groups (*p* > 0.9999). *IFI27* and *RSAD2* showed a contrasting pattern, with markedly higher expression in nT1D compared with both HC (*IFI27*: *p* = 0.0002; *RSAD2*: *p* = 0.0008) and T1D (*IFI27*: *p* = 0.0044; *RSAD2*: *p* = 0.0003), while HC and T1D levels were comparable (*IFI27*: *p* = 0.8308; *RSAD2*: *p* > 0.9999). *ISG15* showed a modest but significant increase in T1D compared with HC (*p* = 0.0205), with no other pairwise comparison reaching significance. In contrast, *IFI44L* and *SIGLEC1* did not differ significantly across any group comparison (all *p* ≥ 0.19 and *p* ≥ 0.29, respectively) (Figure 3).

### 3.6. Associations with Peripheral Blood-Cell Composition

Post-hoc Spearman correlation analyses were performed to explore associations between target transcript expression and leukocyte differential counts in both diabetic cohorts and, for established T1D, lymphocyte subpopulations assessed by flow cytometry. Most correlations did not reach nominal *p* < 0.05. Nominal associations were observed between *FOXP3* transcript abundance and lymphocyte percentage in both new-onset T1D (rho = 0.305, *p* = 0.0418) and established T1D (rho = 0.619, *p* < 0.0001). Additional nominal associations were observed between *FOXP3* and monocyte percentage in new-onset T1D, and between *FOXP3* and neutrophil and CD4+ T-cell percentages in established T1D. *TLR3* was nominally associated with CD4+ T-cell percentage and the CD4/CD8 ratio in established T1D, while *IFIT1* showed nominal associations with lymphocyte percentage in new-onset T1D and with NK-cell percentage in established T1D. Because these were exploratory post-hoc analyses involving multiple comparisons and no multiplicity correction was applied across the correlation panel, all *p*-values should be considered nominal. These associations are therefore hypothesis-generating and should not be interpreted as evidence of cell-specific regulation. Complete results are reported in Supplementary Table S3.

### 3.7. Adjusted and Robustness Analyses

Potential confounding by age and sex was explored within the diabetic cohorts. No association between age or sex and target transcript expression remained statistically significant after FDR correction (Supplementary Table S5). In multivariable regression models adjusted for age, sex, and lymphocyte percentage, the differences between established and new-onset T1D for *TLR3*, *TLR7*, *IFI27*, *RSAD2*, and *IFIT1* remained statistically significant and in the same direction as in the unadjusted analyses (Supplementary Table S6). Effect-size estimates with bootstrap 95% confidence intervals are reported in Supplementary Table S7, while minimum detectable effect sizes for the available sample sizes are summarized in Supplementary Table S8.

### 3.8. Sensitivity Analysis Restricted to Autoantibody-Positive New-Onset T1D

To assess whether the inclusion of autoantibody-negative subjects influenced the primary findings, all between-group gene-expression comparisons were repeated after restricting the new-onset T1D cohort to the 40 patients positive for at least one diabetes-associated autoantibody. Exclusion of the six autoantibody-negative patients did not alter the statistical significance of any of the comparisons observed in the primary analysis. Thus, the overall group-specific expression patterns were unchanged in this sensitivity analysis (Supplementary Table S9).

## 4. Discussion

The present study identifies group-specific differences in whole-blood transcript expression of innate immune genes in type 1 diabetes, characterized by increased *TLR3* and *TLR7* expression together with a heterogeneous pattern of expression among the six interferon-stimulated genes examined. Notably, enterovirus-targeted amplification was observed in only one sample under the applied assay conditions. Because the assay lacked an external positive control, a formally established analytical limit of detection, and independent confirmation of the single positive result, these findings cannot be used to reliably establish either the presence or absence of circulating enterovirus infection in the cohort. Accordingly, the observed transcriptional differences should be interpreted independently of any assumption regarding current systemic enterovirus infection.

The higher whole-blood transcript expression of *TLR3* and *TLR7* identifies differences in genes involved in nucleic acid sensing in T1D. These receptors recognize viral or endogenous RNA species and can promote type I interferon production through downstream signaling pathways. However, the ISG profile observed in this study does not indicate a broad or uniformly coordinated interferon response. Instead, individual ISGs showed distinct patterns across the clinical study groups. *IFI27* and *RSAD2* were significantly increased in new-onset T1D compared with both established T1D and healthy controls, indicating that these genes were more highly expressed in the new-onset group at the time of sampling. In contrast, *IFIT1* was increased in established T1D compared with both healthy controls and new-onset T1D, while *ISG15* showed a more limited increase in established T1D only compared with healthy controls. *SIGLEC1* and *IFI44L* did not show significant differences among groups. In this regard, a recent single-cell RNA sequencing study identified a SIGLEC-1-high monocyte subpopulation with a strong interferon signature in recent-onset autoimmune diabetes [55], indicating that SIGLEC1 can mark a biologically relevant, cell-subset-restricted component of the interferon response in this disease. The present study was not designed to detect such subpopulation-specific effects, as it measured transcript abundance in unfractionated whole blood; cell-sorted or single-cell approaches, as used by Guo et al., would be needed to determine whether a SIGLEC1-high subpopulation is present in this cohort.

This pattern suggests that, among the six ISGs examined here, expression is heterogeneous and group-specific rather than reflecting a generalized IFN signature; these six genes were selected as established markers of the type I IFN transcriptional signature; panels of comparable size (5-21 genes, including *IFI27*, *RSAD2*, and *IFI44L*, closely overlapping with the present panel) are used clinically as validated biomarkers of type I IFN activity in other autoimmune diseases [56,57], and this candidate-gene approach cannot characterize the full breadth of the interferon response, which would require a genome-wide or larger unbiased ISG panel. This heterogeneity is consistent with evidence that ISGs are not induced uniformly but follow distinct early, intermediate, and late temporal induction dynamics, governed by transcript stability and negative-feedback regulation [58]. Exploratory within-patient correlation analyses showed nominal positive associations among several of the selected ISGs, particularly in established T1D, showing correlated variation among several transcripts within the measured panel. These correlations do not establish a shared upstream mechanism or temporal sequence (Supplementary Table S4). The increased expression of *IFI27* and *RSAD2* in new-onset disease may reflect differences in antiviral or RNA-sensing-related transcription at clinical onset, whereas the higher *IFIT1* and *ISG15* expression observed in established T1D may reflect persistent low-grade immune stimulation, chronic inflammatory remodeling, or treatment- and disease-duration-related immune adaptation; as this study is cross-sectional, these two groups represent different individuals rather than the same patients followed over time, and this interpretation should be regarded as hypothesis-generating. Therefore, the data support group-specific differences in the expression of selected interferon-related genes, but do not demonstrate functional or homogeneous activation of the interferon pathway.

The heterogeneous nature of this response, within the six genes examined, is particularly noteworthy. Previous studies have shown that interferon signatures in autoimmune diseases, including T1D, can be heterogeneous and may involve only selected ISGs rather than the entire pathway [24,26,35]. In this context, the lack of significant differences of *SIGLEC1* and *IFI44L* further illustrates the heterogeneous expression pattern observed within the six genes examined; however, this candidate-gene panel cannot determine the overall magnitude or breadth of the systemic interferon response. Overall, the present whole-blood findings indicate a heterogeneous and group-specific pattern among the six ISGs examined, without providing a comprehensive assessment of systemic interferon pathway activity. Enterovirus-targeted amplification was observed in only one sample under the applied assay conditions. Given the analytical limitations of the assay, the absence of amplification in the remaining samples cannot be interpreted as reliable evidence against circulating enterovirus infection. Likewise, these findings do not exclude prior EV exposure, low-level circulating RNA below the assay detection capability, or tissue-restricted infection, including in the pancreas or other compartments [8,12,15]. Tissue-restricted infection could hypothetically contribute to systemic transcriptional or immune changes through cytokine release, antigen presentation, or intermittent exposure to viral components. However, based on the present data, any link between enterovirus and the observed interferon-related gene expression remains indirect.

An alternative hypothesis is that the higher transcript expression of *TLR3* and *TLR7* may be associated with exposure to endogenous RNA released during β-cell stress, apoptosis, or tissue damage. In this model, nucleic acids derived from damaged cells could act as danger-associated molecular patterns and contribute to innate immune signaling in the absence of detectable viral RNA [59,60]. This mechanism may be particularly relevant in new-onset T1D, where β-cell injury and inflammatory processes may be more pronounced in this group [61].

Higher whole-blood *FOXP3* transcript abundance was observed in both diabetic groups. In the post-hoc analysis, *FOXP3* expression was positively associated with lymphocyte percentage in both cohorts and with CD4+ T-cell percentage in established T1D, while inverse associations were observed with selected myeloid-cell proportions. The inclusion of *FOXP3* was exploratory and was intended to assess whether variation in a transcript related to regulatory T-cell biology accompanied the innate immune and IFN-related expression patterns observed in whole blood. These findings suggest that the whole-blood *FOXP3* signal may partly reflect variation in circulating cell composition rather than a purely cell-intrinsic transcriptional change. However, because these correlations were exploratory and corresponding data were not available for healthy controls, they cannot establish the cellular origin or functional significance of the FOXP3 difference (Supplementary Table S3). Several studies have shown that T1D patients may exhibit normal or increased *FOXP3* expression despite impaired Treg function [62,63,64]. This suggests that qualitative defects in Treg activity may be more relevant than quantitative changes.

Furthermore, *FOXP3* expression can be induced in activated conventional T cells without conferring stable regulatory function [44]. Inflammatory conditions, particularly those involving interferon signaling, can influence *FOXP3* stability and Treg plasticity [45,65]. As a result, increased *FOXP3* transcript abundance may be one of several possible correlates of chronic inflammation and altered lymphocyte proportions in T1D, rather than direct evidence of a compensatory regulatory response.

Overall, these findings indicate group-specific differences in whole-blood transcript expression of selected RNA-sensing and interferon-related genes in T1D, while enterovirus-targeted amplification was observed in only one sample under the applied assay conditions. The ISG pattern observed here should not be interpreted as a broad interferon signature, but rather as a heterogeneous, group-specific transcriptional profile restricted to the six genes examined. This distinction is important because it suggests that different interferon-stimulated genes may capture different aspects of immune activation associated with clinical disease status in T1D. As new-onset and established T1D represent different individuals studied at a single time point rather than the same patients followed over time, these differences should be interpreted as group-specific associations rather than evidence of temporal or longitudinal progression.

Future studies integrating longitudinal sampling, serological assessment, and tissue-based viral detection will be necessary to clarify whether these transcriptional changes reflect prior viral exposure, tissue-restricted infection, endogenous RNA-driven inflammation, or a combination of these mechanisms. Recent longitudinal single-cell transcriptomic studies in children followed from early life to seroconversion illustrate the type of approach that could resolve these questions in future work [66].

### Study Limitations

This study has several limitations. First, enterovirus RNA was assessed only in peripheral whole blood using a *5′NCR*-targeted RT-PCR assay, and amplification was observed in only one sample. No external positive control was included, the analytical limit of detection and amplification efficiency were not formally established, and the single assay-positive result was not independently confirmed. Therefore, absence of amplification in the remaining samples cannot reliably exclude low-level circulating EV RNA, while the identity and biological significance of the single positive result remain uncertain. Prior exposure or tissue-restricted persistence in pancreatic, gut-associated, or lymphoid compartments also cannot be excluded [8,12,15]. Pancreatic or islet tissue was not available, and any relationship between EV exposure and the observed transcriptional differences remains indirect.

Second, EV serology and circulating type I IFN protein levels were not assessed because suitable serum or plasma samples were unavailable. Retrospective EV serology is also limited by cross-reactivity, high background seroprevalence, and the difficulty of assigning serotype-specific exposure [14,67]. In addition, the study relied on transcript-level measurements without protein-level or functional validation. Although ISG transcriptional panels are widely used as surrogate biomarkers of type I IFN activity [55,56], mRNA abundance does not necessarily reflect protein expression or biological activity.

Third, gene expression was measured in unfractionated whole blood. Most post-hoc correlations with leukocyte composition were not statistically significant, although nominal associations were observed for *FOXP3*, *TLR3*, and *IFIT1* with selected leukocyte subsets. These exploratory findings do not exclude confounding by blood-cell composition, and leukocyte differential data were not available for healthy controls (Supplementary Table S3).

Fourth, the cross-sectional design does not permit temporal or longitudinal interpretation. New-onset and established T1D represent different individuals assessed at a single time point, and the observed expression patterns should therefore be interpreted as group-specific associations rather than evidence of disease progression. The new-onset and established T1D groups differed in age and sex distribution; however, no association between these variables and target transcript expression remained statistically significant after correction for multiple comparisons (Supplementary Table S5). In addition, multivariable adjustment for age, sex, and lymphocyte percentage did not materially alter the significant differences observed between established and new-onset T1D for *TLR3*, *TLR7*, *IFI27*, *RSAD2*, and *IFIT1* (Supplementary Table S6). Nevertheless, residual confounding cannot be excluded, particularly because equivalent adjustment could not be performed for comparisons involving healthy controls. Information on the precise timing of blood collection relative to insulin initiation, metabolic stabilization, and DKA treatment was not available in a sufficiently standardized form for the entire cohort. Data on recent infections, vaccinations, and concomitant medications other than insulin were also not systematically available. The available sample size and incomplete availability of several clinical covariates also limited reliable post-hoc stratification into additional clinical subgroups. Nevertheless, restricting the new-onset T1D cohort to autoantibody-positive patients did not alter the statistical significance of the primary between-group comparisons (Supplementary Table S9). In addition, systematic genetic testing for monogenic diabetes was not performed in all autoantibody-negative patients; therefore, alternative diabetes forms cannot be excluded with absolute certainty. Finally, this was an exploratory candidate-gene study. Although FDR correction was applied across the complete gene panel and all reported significant comparisons remained significant (Supplementary Table S2), no formal a priori sample-size calculation was performed. The study was powered mainly to detect medium-to-large effects, while smaller effects could not be reliably excluded (Supplementary Table S8). Effect sizes, expressed as rank-biserial correlations with bootstrap 95% confidence intervals, are reported in Supplementary Table S7. *GAPDH* was used as the sole reference gene. Although raw *GAPDH* Ct values did not differ significantly among the study groups, multi-reference-gene normalization was not performed and should be considered in future studies.

## Figures and Tables

**Table 1:** Primers and Probes used for amplification.

Target	Primers		Probes
*GAPDH*	PF	5′-CGAGATCCCTCCAAAATCAA-3′	6FAM-TGGAGAAGGCTGGGGCTCAT-TAMRA
	PR	5′-TTCACACCCATGACGAACAT-3′	
*TLR3*	PF	5′-TGGTTGGGCCACCTAGAAGTA-3′	6FAM-ACCTGGGCCTTAATGAAATTGGGCAA-TAMRA
	PR	5′-TCTCCATTCCTGGCCTGTG-3′	
*TLR7*	PF	5′-TTACCTGGATGGAAACCAGCTACT-3′	6FAM-AGATACCGCAGGGCCTCCCGC-TAMRA
	PR	5′-TCAAGGCTGAGAAGCTGTAAGCTA-3′	
*TLR8*	PF	5′-CTGCGCTACCACCTTGAAGA-3′	FAM-GCCGAGACAAAAACGTTCTCCTTTGT-BHQ1
	PR	5′-GGTCCCAATCCCTCTCCTCT-3′	
*FOXP3*	PF	5′-TCACCTACGCCACGCTCAT-3′	6FAM-CTGGGCCATCCTGGA-MGB
	PR	5′-ATTGAGTGTCCGCTGCTTCTC-3′	
*5′NCR*	PF	5′-CCCTGAATGCGGCTAATCC-3′	JOE-AACCGACTACTTTGGGTGTCCGTGTTTC-BHQ2
	PR	5′-ATTGTCACCATAAGCAGCCA-3′	

**Table 2:** Clinical characteristics of the enrolled subjects.

Characteristic	T1D (*n* = 48)	nT1D (*n* = 46)	HC–IFN-I Panel (*n* = 35)	HC–TLR/FOXP3 Panel (*n* = 25)
Age, years: Median (IQR)	11.84 (9.59–13.45)	9.94 (5.95–12.54)	11.56 (7.25–13.08)	11.00 (8.00–13.00)
Sex, M/F, *n*	31/17	19/27	21/14	13/12
BMI: Median (IQR 25–75%)	18.75 (17.05–21.73)	16.84 (15.97–19.73)	N/A (non-diabetic)	N/A (non-diabetic)
BMI SDS (WHO): Median (IQR 25–75%)	0.36 (−0.03–1.04)	0.18 (−0.42–0.86)	N/A (non-diabetic)	N/A (non-diabetic)
HbA1c at original diagnosis: Median (IQR 25–75%)	11.25 (9.98–11.80)	11.60 (9.88–13.08)	N/A (non-diabetic)	N/A (non-diabetic)
HbA1c near blood collection (last clinic visit): Median (IQR 25–75%)	6.90 (6.47–7.25)	N/A (new-onset)	N/A (non-diabetic)	N/A (non-diabetic)
Disease duration, years: Median (IQR)	4.36 (2.16–7.53)	N/A (new-onset)	N/A	N/A
Ongoing therapy: Insulin pen, *n* (%)	9 (18.75)	N/A (new-onset, insulin not yet established)	N/A	N/A
Ongoing therapy: Insulin pump, *n* (%)	39 (81.25)	N/A (new-onset, insulin not yet established)	N/A	N/A
Comorbidities: Celiac disease, *n* (%)	5 (10.4)	6 (12.8)	0 (exclusion criterion)	0 (exclusion criterion)
Comorbidities: Thyroid disorders, *n* (%)	6 (12.5)	1 (2.1)	0 (exclusion criterion)	0 (exclusion criterion)
Laboratory findings	See above	See above	No laboratory abnormalities at recruitment (exclusion criterion)	No laboratory abnormalities at recruitment (exclusion criterion)

T1D = Type 1 Diabetes, nT1D= new-onset Type 1 Diabetes, HC=Healthy Controls, *n* = number, IQR = interquartile range. The T1D and nT1D groups differed significantly in age (median 11.84 vs. 9.94 years, Mann–Whitney *p* = 0.021) and sex distribution (chi-square *p* = 0.040). No association between age or sex and target transcript expression remained statistically significant after correction for multiple comparisons; full results are reported in Supplementary Table S5. Disease duration in the T1D group was median 4.36 years (IQR 2.16–7.53). HbA1c is reported at original diagnosis above; HbA1c near the time of blood collection (last clinic visit) in the T1D group was median 6.90% (IQR 6.47–7.25).

## Data Availability

The data presented in this study are available on request from the corresponding author. The data are not publicly available due to privacy restrictions.

## Supplementary Materials

The following supporting information can be downloaded at https://www.mdpi.com/article/10.3390/genes17091065/s1. Table S1: Sample accounting; Table S2: Benjamini–Hochberg FDR correction across the complete gene panel; Table S3: Correlations between target transcript expression and leukocyte differential counts and lymphocyte subpopulations; Table S4: Exploratory correlations among selected interferon-stimulated gene transcripts; Table S5: Associations of age and sex with target expression; Table S6: Multivariable regression analyses; Table S7: Effect sizes with 95% confidence intervals; Table S8: Minimum detectable effect sizes and retrospective power analysis; Table S9: Sensitivity analysis restricted to autoantibody-positive new-onset T1D patients.
