# Supplementary material for: Selective Whole-Blood Expression of Interferon-Stimulated Genes and TLR3/TLR7 in Type 1 Diabetes with Minimal Enterovirus-Targeted Amplification in Whole Blood
Source: Genes (Basel). 2026 Sep 2;17(9):1065. doi: 10.3390/genes17091065 (PMC13606364; doi:10.3390/genes17091065)
Supplement: Supplementary file 1 [file genes-17-01065-s001.zip › genes-4388797-supplementary.pdf]

Supplementary Materials

Table S1. Evaluable sample size per target gene and cohort, relative to the nominal recruited cohort size (Table 2). Differences between nominal and evaluable n reflect samples excluded due to inadequate RNA yield, amplification failure, or missing data for that specific target.

Gene target	T1D evaluable (*n*)	T1D excluded/missing	nT1D evaluable (*n*)	nT1D excluded/missing	HC evaluable (*n*)	HC excluded/missing	HC nominal cohort (*n*)
TLR3	47	1	45	1	25	0	25
TLR7	47	1	45	1	25	0	25
TLR8	46	2	45	1	25	0	25
IFI44L	48	0	46	0	35	0	35
ISG15	48	0	46	0	35	0	35
IFIT1	48	0	46	0	35	0	35
IFI27	48	0	46	0	33	2	35
RSAD2	48	0	46	0	35	0	35
SIGLEC1	48	0	46	0	35	0	35
FOXP3	46	2	45	1	25	0	25

Table S2. Benjamini-Hochberg false discovery rate correction across the complete panel of 30 pairwise comparisons (10 targets x 3 group comparisons). FDR correction was performed using the complete numerical set of exact Dunn's multiple-comparison p-values. All 14 comparisons significant at nominal $p<0.05$ remained significant after FDR correction.

**Gene**	**Comparison**	***p* (raw, Dunn's)**	***p*_FDR**	**Significant (FDR<0.05)**
TLR3	T1D vs HC	<0.0001	0.001	Yes
TLR3	T1D vs nT1D	0.0001	0.001	Yes
TLR3	nT1D vs HC	0.0933	0.1866	No
TLR7	T1D vs HC	<0.0001	0.001	Yes
TLR7	T1D vs nT1D	0.0038	0.0142	Yes
TLR7	nT1D vs HC	0.0006	0.003	Yes
TLR8	T1D vs nT1D	>0.9999	0.9999	No
TLR8	T1D vs HC	0.9173	0.9999	No
TLR8	nT1D vs HC	0.5950	0.7437	No
FOXP3	T1D vs HC	0.0071	0.0192	Yes
FOXP3	nT1D vs HC	0.0142	0.0328	Yes
FOXP3	T1D vs nT1D	>0.9999	0.9999	No
IFIT1	T1D vs nT1D	0.0077	0.0192	Yes
IFIT1	T1D vs HC	0.0073	0.0192	Yes
IFIT1	nT1D vs HC	>0.9999	0.9999	No

IFI27	nT1D vs HC	0.0002	0.0015	Yes
IFI27	nT1D vs T1D	0.0044	0.0147	Yes
IFI27	HC vs T1D	0.8308	0.997	No
RSAD2	nT1D vs HC	0.0008	0.0034	Yes
RSAD2	nT1D vs T1D	0.0003	0.0018	Yes
RSAD2	HC vs T1D	>0.9999	0.9999	No
ISG15	T1D vs HC	0.0205	0.0439	Yes
ISG15	T1D vs nT1D	0.8286	0.9999	No
ISG15	nT1D vs HC	0.2651	0.4418	No
IFI44L	T1D vs HC	>0.9999	0.9999	No
IFI44L	T1D vs nT1D	0.1907	0.3576	No
IFI44L	nT1D vs HC	0.2227	0.3930	No
SIGLEC1	T1D vs HC	0.4877	0.7316	No
SIGLEC1	T1D vs nT1D	>0.9999	0.9999	No
SIGLEC1	nT1D vs HC	0.2948	0.4655	No

Table S3. Exploratory correlation between target gene expression and leukocyte differential counts (neutrophils, lymphocytes, monocytes, eosinophils, basophils), and, for established T1D, lymphocyte subpopulations by flow cytometry (CD3+, CD4+, CD8+, CD19+, NK, CD4/CD8 ratio). Leukocyte differential data were not available for the healthy control group. This analysis is exploratory and involves multiple statistical comparisons. *P*-values are reported as nominal and were not corrected for multiplicity; therefore, these associations should be considered hypothesis-generating and should not be interpreted as confirmatory evidence of cell-specific regulation.

**Cohort**	**Gene**	**Cell parameter**	***n***	**rho**	***p***	**Nominal *p*<0.05 (uncorrected)**
**New-onset T1D**	*TLR3*	NEUTROPHILS	45	-0.011	0.9418	No
**New-onset T1D**	*TLR3*	LYMPHOCYTES	45	-0.006	0.9712	No
**New-onset T1D**	*TLR3*	MONOCYTES	45	0.078	0.6091	No
**New-onset T1D**	*TLR3*	EOSINOPHILS	45	0.094	0.5378	No
**New-onset T1D**	*TLR3*	BASOPHILS	45	-0.047	0.7591	No
**New-onset T1D**	*TLR7*	NEUTROPHILS	45	-0.082	0.5928	No
**New-onset T1D**	*TLR7*	LYMPHOCYTES	45	0.059	0.702	No
**New-onset T1D**	*TLR7*	MONOCYTES	45	0.069	0.6517	No
**New-onset T1D**	*TLR7*	EOSINOPHILS	45	0.17	0.2653	No
**New-onset T1D**	*TLR7*	BASOPHILS	45	0.045	0.7703	No
**New-onset T1D**	*FOXP3*	NEUTROPHILS	45	-0.268	0.0754	No
**New-onset T1D**	*FOXP3*	LYMPHOCYTES	45	0.305	0.0418	Yes
**New-onset T1D**	*FOXP3*	MONOCYTES	45	-0.392	0.0077	Yes
**New-onset T1D**	*FOXP3*	EOSINOPHILS	45	-0.009	0.9534	No

**New-onset T1D**	*FOXP3*	BASOPHILS	45	0.038	0.8033	No
**New-onset T1D**	*IFI27*	NEUTROPHILS	46	-0.068	0.6536	No
**New-onset T1D**	*IFI27*	LYMPHOCYTES	46	0.035	0.8175	No
**New-onset T1D**	*IFI27*	MONOCYTES	46	0.24	0.1082	No
**New-onset T1D**	*IFI27*	EOSINOPHILS	46	0.147	0.3313	No
**New-onset T1D**	*IFI27*	BASOPHILS	46	0.104	0.4911	No
**New-onset T1D**	*ISG15*	NEUTROPHILS	46	0.213	0.1559	No
**New-onset T1D**	*ISG15*	LYMPHOCYTES	46	-0.244	0.1027	No
**New-onset T1D**	*ISG15*	MONOCYTES	46	-0.007	0.9627	No
**New-onset T1D**	*ISG15*	EOSINOPHILS	46	0.209	0.1633	No
**New-onset T1D**	*ISG15*	BASOPHILS	46	0.202	0.1773	No
**New-onset T1D**	*RSAD2*	NEUTROPHILS	46	0.146	0.333	No
**New-onset T1D**	*RSAD2*	LYMPHOCYTES	46	-0.137	0.3636	No
**New-onset T1D**	*RSAD2*	MONOCYTES	46	-0.173	0.2494	No
**New-onset T1D**	*RSAD2*	EOSINOPHILS	46	-0.033	0.8264	No
**New-onset T1D**	*RSAD2*	BASOPHILS	46	0.044	0.7706	No
**New-onset T1D**	*IFIT1*	NEUTROPHILS	46	0.277	0.0625	No
**New-onset T1D**	*IFIT1*	LYMPHOCYTES	46	-0.341	0.0205	Yes
**New-onset T1D**	*IFIT1*	MONOCYTES	46	0.015	0.9214	No
**New-onset T1D**	*IFIT1*	EOSINOPHILS	46	0.153	0.3089	No
**New-onset T1D**	*IFIT1*	BASOPHILS	46	0.113	0.4549	No
**Established T1D**	*TLR3*	NEUTROPHILS	46	-0.085	0.575	No
**Established T1D**	*TLR3*	LYMPHOCYTES	46	0.14	0.3536	No
**Established T1D**	*TLR3*	MONOCYTES	46	0.03	0.8418	No
**Established T1D**	*TLR3*	EOSINOPHILS	46	0.055	0.7149	No
**Established T1D**	*TLR3*	BASOPHILS	46	-0.114	0.4516	No
**Established T1D**	*TLR3*	T (CD3)	44	0.161	0.2951	No
**Established T1D**	*TLR3*	T helper (CD3+/CD4+)	44	-0.418	0.0047	Yes
**Established T1D**	*TLR3*	Cytotoxic T cells (CD3+/CD8+)	44	0.294	0.0524	No
**Established T1D**	*TLR3*	B cells (CD19+)	44	-0.261	0.0865	No
**Established T1D**	*TLR3*	NK (CD16/+CD56+)	44	0.095	0.5417	No

**Established T1D**	*TLR3*	CD4/CD8 Ratio	44	-0.462	0.0016	Yes
**Established T1D**	*TLR7*	NEUTROPHILS	46	0.049	0.7472	No
**Established T1D**	*TLR7*	LYMPHOCYTES	46	-0.119	0.4327	No
**Established T1D**	*TLR7*	MONOCYTES	46	0.108	0.4749	No
**Established T1D**	*TLR7*	EOSINOPHILS	46	0.052	0.7296	No
**Established T1D**	*TLR7*	BASOPHILS	46	-0.001	0.9967	No
**Established T1D**	*TLR7*	T cells (CD3+)	44	0.078	0.6148	No
**Established T1D**	*TLR7*	T helper (CD3+/CD4+)	44	-0.046	0.7651	No
**Established T1D**	*TLR7*	Cytotoxic T cells (CD3+/CD8+)	44	0.016	0.9188	No
**Established T1D**	*TLR7*	B cells (CD19+)	44	0.082	0.5967	No
**Established T1D**	*TLR7*	NK (CD16+/CD56+)	44	-0.191	0.2146	No
**Established T1D**	*TLR7*	CD4/CD8 Ratio	44	-0.052	0.7356	No
**Established T1D**	*FOXP3*	NEUTROPHILS	45	-0.481	0.0008	Yes
**Established T1D**	*FOXP3*	LYMPHOCYTES	45	0.619	< 0.0001	Yes
**Established T1D**	*FOXP3*	MONOCYTES	45	-0.216	0.1541	No
**Established T1D**	*FOXP3*	EOSINOPHILS	45	-0.007	0.9623	No
**Established T1D**	*FOXP3*	BASOPHILS	45	-0.082	0.5929	No
**Established T1D**	*FOXP3*	T cells (CD3+)	43	-0.047	0.7627	No
**Established T1D**	*FOXP3*	T helper (CD3+/CD4+)	43	0.396	0.0085	Yes
**Established T1D**	*FOXP3*	Cytotoxic T cells (CD3+/CD8+)	43	-0.111	0.4784	No
**Established T1D**	*FOXP3*	B cells (CD19+)	43	0.055	0.725	No
**Established T1D**	*FOXP3*	NK (CD16+/CD56+)	43	-0.047	0.7643	No
**Established T1D**	*FOXP3*	CD4/CD8 Ratio	43	0.217	0.1615	No
**Established T1D**	*IFI27*	NEUTROPHILS	47	-0.265	0.0713	No
**Established T1D**	*IFI27*	LYMPHOCYTES	47	0.04	0.7912	No
**Established T1D**	*IFI27*	MONOCYTES	47	0.155	0.2997	No
**Established T1D**	*IFI27*	EOSINOPHILS	47	0.221	0.1361	No
**Established T1D**	*IFI27*	BASOPHILS	47	0.132	0.3756	No
**Established T1D**	*IFI27*	T cells (CD3+)	45	0.066	0.6678	No
**Established T1D**	*IFI27*	T helper (CD3+/CD4+)	45	-0.044	0.7745	No

**Established T1D**	*IFI27*	Cytotoxic T cells (CD3+/CD8+)	45	0.108	0.4796	No
**Established T1D**	*IFI27*	B cells (CD19+)	45	-0.139	0.3642	No
**Established T1D**	*IFI27*	NK (CD16+/CD56+)	45	0.056	0.7159	No
**Established T1D**	*IFI27*	CD4/CD8 Ratio	45	-0.104	0.4961	No
**Established T1D**	*ISG15*	NEUTROPHILS	47	-0.087	0.56	No
**Established T1D**	*ISG15*	LYMPHOCYTES	47	-0.196	0.1864	No
**Established T1D**	*ISG15*	MONOCYTES	47	0.19	0.2018	No
**Established T1D**	*ISG15*	EOSINOPHILS	47	0.189	0.2024	No
**Established T1D**	*ISG15*	BASOPHILS	47	0.256	0.0822	No
**Established T1D**	*ISG15*	T cells (CD3+)	45	0.189	0.2125	No
**Established T1D**	*ISG15*	T helper (CD3+/CD4+)	45	-0.015	0.9208	No
**Established T1D**	*ISG15*	Cytotoxic T cells (CD3+/CD8+)	45	0.063	0.6813	No
**Established T1D**	*ISG15*	B cells (CD19+)	45	-0.148	0.3319	No
**Established T1D**	*ISG15*	NK (CD16+/CD56+)	45	-0.11	0.4705	No
**Established T1D**	*ISG15*	CD4/CD8 Ratio	45	-0.039	0.798	No
**Established T1D**	*RSAD2*	NEUTROPHILS	47	-0.031	0.8365	No
**Established T1D**	*RSAD2*	LYMPHOCYTES	47	-0.28	0.0569	No
**Established T1D**	*RSAD2*	MONOCYTES	47	0.165	0.268	No
**Established T1D**	*RSAD2*	EOSINOPHILS	47	0.181	0.2238	No
**Established T1D**	*RSAD2*	BASOPHILS	47	0.131	0.3797	No
**Established T1D**	*RSAD2*	T cells (CD3+)	45	0.08	0.6033	No
**Established T1D**	*RSAD2*	T helper (CD3+/CD4+)	45	-0.043	0.7812	No
**Established T1D**	*RSAD2*	Cytotoxic T cells (CD3+/CD8+)	45	0.017	0.9129	No
**Established T1D**	*RSAD2*	B cells (CD19+)	45	0.027	0.8616	No
**Established T1D**	*RSAD2*	NK (CD16+/CD56+)	45	-0.158	0.3012	No
**Established T1D**	*RSAD2*	CD4/CD8 Ratio	45	0.001	0.9935	No
**Established T1D**	*IFIT1*	NEUTROPHILS	47	0.013	0.9327	No
**Established T1D**	*IFIT1*	LYMPHOCYTES	47	-0.19	0.2003	No
**Established T1D**	*IFIT1*	MONOCYTES	47	0.03	0.8414	No
**Established T1D**	*IFIT1*	EOSINOPHILS	47	-0.023	0.8773	No

**Established T1D**	*IFIT1*	BASOPHILS	47	0.148	0.3213	No
**Established T1D**	*IFIT1*	T cells (CD3+)	45	0.11	0.4733	No
**Established T1D**	*IFIT1*	T helper (CD3+/CD4+)	45	0.072	0.6384	No
**Established T1D**	*IFIT1*	Cytotoxic T cells (CD3+/CD8+)	45	-0.13	0.3931	No
**Established T1D**	*IFIT1*	B cells (CD19+)	45	0.027	0.8609	No
**Established T1D**	*IFIT1*	NK (CD16+/CD56+)	45	-0.295	0.049	Yes
**Established T1D**	*IFIT1*	CD4/CD8 Ratio	45	0.079	0.6038	No

Table S4. Exploratory correlations among *IFI27, RSAD2, IFIT1*, and *ISG15* transcript levels within the new-onset and established T1D cohorts. This analysis is exploratory; *p*-values are reported as nominal and were not corrected for multiplicity. The observed correlations should be considered hypothesis-generating associations among the selected transcripts and should not be interpreted as evidence of coordinated pathway activation or a shared upstream regulatory mechanism.

**Cohort**	**Gene pair**	***n***	**rho**	***p***	**Nominal *p*<0.05 (uncorrected)**
**New-onset T1D**	*IFI27 vs RSAD2*	46	0.513	0.00027	Yes
**New-onset T1D**	*IFI27 vs IFIT1*	46	-0.028	0.8549	No
**New-onset T1D**	*IFI27 vs ISG15*	46	0.404	0.00536	Yes
**New-onset T1D**	*RSAD2 vs IFIT1*	46	0.089	0.55684	No
**New-onset T1D**	*RSAD2 vs ISG15*	46	0.557	6e-05	Yes
**New-onset T1D**	*IFIT1 vs ISG15*	46	0.481	0.00071	Yes
**Established T1D**	*IFI27 vs RSAD2*	48	0.531	0.0001	Yes
**Established T1D**	*IFI27 vs IFIT1*	48	0.409	0.0039	Yes
**Established T1D**	*IFI27 vs ISG15*	48	0.582	1e-05	Yes
**Established T1D**	*RSAD2 vs IFIT1*	48	0.688	< 0.0001	Yes
**Established T1D**	*RSAD2 vs ISG15*	48	0.904	< 0.0001	Yes
**Established T1D**	*IFIT1 vs ISG15*	48	0.688	< 0.0001	Yes

Table S5. Association between age (Spearman correlation) and sex (Mann-Whitney U test) and expression of each target gene, within the new-onset and established T1D cohorts (28 comparisons total). One nominal association (sex vs. *TLR7*, established T1D, *p*=0.0076) did not remain significant after Benjamini-Hochberg FDR correction across all 28 comparisons (FDR-adjusted *p*=0.21), consistent with the ~1.4 false positives expected by chance at this number of tests. No association between age or sex and target transcript expression remained statistically significant after FDR correction; however, residual confounding cannot be completely excluded.

**Cohort**	**Gene**	**Test**	***n***	**Statistic**	***p***	***p*_FDR**	**Significant (FDR<0.05)**
**New-onset T1D**	*TLR3*	Age (Spearman rho)	45	rho=+0.202	0.1828	0.7297	No
**New-onset T1D**	*TLR3*	Sex (Mann-Whitney M vs F)	19M/26F	-	0.9176	0.9516	No

**New-onset T1D**	*TLR7*	Age (Spearman rho)	45	rho=+0.219	0.1474	0.6879	No
**New-onset T1D**	*TLR7*	Sex (Mann-Whitney M vs F)	19M/26F	-	0.352	0.7297	No
**New-onset T1D**	*FOXP3*	Age (Spearman rho)	45	rho=+0.095	0.5359	0.7898	No
**New-onset T1D**	*FOXP3*	Sex (Mann-Whitney M vs F)	19M/26F	-	0.7046	0.822	No
**New-onset T1D**	*IFI27*	Age (Spearman rho)	46	rho=-0.289	0.0517	0.4319	No
**New-onset T1D**	*IFI27*	Sex (Mann-Whitney M vs F)	19M/27F	-	0.8759	0.9433	No
**New-onset T1D**	*ISG15*	Age (Spearman rho)	46	rho=-0.108	0.474	0.7898	No
**New-onset T1D**	*ISG15*	Sex (Mann-Whitney M vs F)	19M/27F	-	0.7379	0.8264	No
**New-onset T1D**	*RSAD2*	Age (Spearman rho)	46	rho=-0.131	0.3871	0.7297	No
**New-onset T1D**	*RSAD2*	Sex (Mann-Whitney M vs F)	19M/27F	-	0.577	0.8078	No
**New-onset T1D**	*IFIT1*	Age (Spearman rho)	46	rho=+0.130	0.3909	0.7297	No
**New-onset T1D**	*IFIT1*	Sex (Mann-Whitney M vs F)	19M/27F	-	0.3899	0.7297	No
**Established T1D**	*TLR3*	Age (Spearman rho)	47	rho=+0.156	0.2958	0.7297	No
**Established T1D**	*TLR3*	Sex (Mann-Whitney M vs F)	30M/17F	-	0.3699	0.7297	No
**Established T1D**	*TLR7*	Age (Spearman rho)	47	rho=-0.008	0.9569	0.9569	No
**Established T1D**	*TLR7*	Sex (Mann-Whitney M vs F)	30M/17F	-	0.0076	0.2137	No
**Established T1D**	*FOXP3*	Age (Spearman rho)	46	rho=+0.071	0.6407	0.8112	No
**Established T1D**	*FOXP3*	Sex (Mann-Whitney M vs F)	29M/17F	-	0.649	0.8112	No
**Established T1D**	*IFI27*	Age (Spearman rho)	48	rho=-0.104	0.4811	0.7898	No
**Established T1D**	*IFI27*	Sex (Mann-Whitney M vs F)	31M/17F	-	0.6664	0.8112	No
**Established T1D**	*ISG15*	Age (Spearman rho)	48	rho=-0.149	0.3129	0.7297	No

**Established T1D**	*ISG15*	Sex (Mann-Whitney M vs F)	31M/17F	-	0.2192	0.7297	No
**Established T1D**	*RSAD2*	Age (Spearman rho)	48	rho=-0.267	0.0663	0.4319	No
**Established T1D**	*RSAD2*	Sex (Mann-Whitney M vs F)	31M/17F	-	0.0771	0.4319	No
**Established T1D**	*IFIT1*	Age (Spearman rho)	48	rho=-0.093	0.5299	0.7898	No
**Established T1D**	*IFIT1*	Sex (Mann-Whitney M vs F)	31M/17F	-	0.0771	0.4319	No

Table S6. Multivariable linear regression of log-transformed RQ on group (established T1D vs. new-onset T1D), adjusted for age, sex, and lymphocyte percentage. This model could only be constructed for the T1D vs. nT1D comparison, as age, sex, and leukocyte differential data were not available at the individual level for the healthy control group. All group effects that were significant in the unadjusted analysis (*TLR3, TLR7, IFI27, RSAD2, IFIT1*) remained significant with the same direction of effect after adjustment; *FOXP3* and *ISG15* remained non-significant, consistent with the unadjusted analysis.

Gene	*n*	Beta (T1D vs nT1D, log-RQ)	CI low	CI high	*p*	Significant (*p*<0.05)
TLR3	91	0.516	0.191	0.84	0.0022	Yes
TLR7	91	0.294	0.046	0.542	0.0207	Yes
FOXP3	90	0.285	-0.091	0.662	0.1358	No
IFI27	93	-0.678	-1.239	-0.118	0.0182	Yes
ISG15	93	0.129	-0.311	0.57	0.5609	No
RSAD2	93	-0.728	-1.224	-0.232	0.0045	Yes
IFIT1	93	0.76	0.285	1.236	0.002	Yes

Table S7. Effect sizes (rank-biserial correlation, r, derived from the Mann–Whitney U statistic) with bootstrap 95% confidence intervals based on 2000 resamples for all pairwise comparisons of *TLR3, TLR7, TLR8, IFI44L, ISG15, IFIT1, IFI27, RSAD2, SIGLEC1,* and *FOXP3*, using raw per-subject RQ data from all three groups. The absolute value of r may be interpreted analogously to a correlation coefficient, with values of approximately 0.1, 0.3, and 0.5 indicating small, medium, and large effects, respectively. Because confidence intervals were obtained from unadjusted pairwise bootstrap analyses, they may not coincide exactly with significance based on Dunn's multiple-comparison test and FDR-adjusted p-values reported in Table S2.

Gene	Comparison	*n1*	*n2*	Rank-biserial r	95% CI low	95% CI high
TLR3	T1D vs HC	47	25	-0.813	-0.929	-0.675
TLR3	T1D vs nT1D	47	45	-0.496	-0.696	-0.28
TLR3	nT1D vs HC	45	25	-0.308	-0.548	-0.035
TLR7	T1D vs HC	47	25	-0.894	-0.973	-0.782
TLR7	T1D vs nT1D	47	45	-0.407	-0.615	-0.173
TLR7	nT1D vs HC	45	25	-0.57	-0.774	-0.353
TLR8	T1D vs HC	46	25	-0.12	-0.397	0.148
TLR8	T1D vs nT1D	46	45	0.023	-0.237	0.269
TLR8	nT1D vs HC	45	25	-0.214	-0.483	0.06
IFI44L	T1D vs HC	48	35	0.008	-0.251	0.248
IFI44L	T1D vs nT1D	48	46	0.222	-0.016	0.458

IFI44L	nT1D vs HC	46	35	-0.27	-0.507	-0.031
ISG15	T1D vs HC	48	35	-0.345	-0.573	-0.104
ISG15	T1D vs nT1D	48	46	-0.13	-0.355	0.108
ISG15	nT1D vs HC	46	35	-0.222	-0.462	0.037
IFIT1	T1D vs HC	48	35	-0.43	-0.633	-0.187
IFIT1	T1D vs nT1D	48	46	-0.345	-0.554	-0.112
IFIT1	nT1D vs HC	46	35	0.037	-0.207	0.288
IFI27	T1D vs HC	48	33	-0.131	-0.385	0.123
IFI27	T1D vs nT1D	48	46	0.389	0.172	0.589
IFI27	nT1D vs HC	46	33	-0.547	-0.74	-0.32
RSAD2	T1D vs HC	48	35	0.018	-0.238	0.276
RSAD2	T1D vs nT1D	48	46	0.472	0.245	0.681
RSAD2	nT1D vs HC	46	35	-0.525	-0.732	-0.297
SIGLEC1	T1D vs HC	48	35	-0.186	-0.424	0.056
SIGLEC1	T1D vs nT1D	48	46	0.053	-0.18	0.283
SIGLEC1	nT1D vs HC	46	35	-0.219	-0.462	0.042
FOXP3	T1D vs HC	46	25	-0.45	-0.704	-0.184
FOXP3	T1D vs nT1D	46	45	-0.022	-0.262	0.217
FOXP3	nT1D vs HC	45	25	-0.396	-0.644	-0.122

Table S8. Minimum detectable effect size (Cohen's d) at 80% power and alpha=0.05 (two-sided), based on the nominal cohort sizes reported in Table 2 for each comparison (actual per-gene n varied slightly due to missing data, as noted throughout Results). This exploratory study was formally powered to detect medium-to-large effect sizes (d approximately 0.58-0.71) with 80% probability; smaller effects could not be reliably excluded. A formal a priori power/sample-size calculation was not performed prior to data collection.

**Comparison**	***n1***	***n2***	**Minimum detectable Cohen's d (80% power, alpha=0.05)**
**T1D (*n*=48) vs HC - TLR/FOXP3 panel (*n*=25)**	48	25	0.701
**T1D (*n*=48) vs HC - IFN-I panel (*n*=35)**	48	35	0.63
**T1D (*n*=48) vs nT1D (*n*=46)**	48	46	0.584
**nT1D (*n*=46) vs HC - TLR/FOXP3 panel (*n*=25)**	46	25	0.706
**nT1D (*n*=46) vs HC - IFN-I panel (*n*=35)**	46	35	0.636

Table S9. Sensitivity analysis restricted to autoantibody-positive new-onset T1D patients.
Primary between-group comparisons were repeated after excluding the six autoantibody-negative patients from the new-onset T1D cohort. The analysis therefore included autoantibody-positive new-onset T1D patients (n = 40), established T1D patients, and healthy controls. Statistical comparisons were performed using the Kruskal–Wallis test followed by Dunn's multiple comparisons test, consistent with the primary analysis. Exclusion of the autoantibody-negative new-onset T1D patients did not alter the statistical significance of any of the primary pairwise comparisons.

**Gene**	**Comparison**	**Dunn's *p*-value**	**Significant (*p* < 0.05)**	**Primary analysis significance**
TLR3	T1D vs HC	< 0.0001	Yes	Yes
TLR3	T1D vs autoantibody-positive nT1D	< 0.0001	Yes	Yes
TLR3	autoantibody-positive nT1D vs HC	0.1934	No	No
TLR7	T1D vs HC	< 0.0001	Yes	Yes

TLR7	T1D vs autoantibody-positive nT1D	0.0019	Yes	Yes
TLR7	autoantibody-positive nT1D vs HC	0.0013	Yes	Yes
TLR8	T1D vs HC	0.9215	No	No
TLR8	T1D vs autoantibody-positive nT1D	>0.9999	No	No
TLR8	autoantibody-positive nT1D vs HC	0.6352	No	No
FOXP3	T1D vs HC	0.0062	Yes	Yes
FOXP3	T1D vs autoantibody-positive nT1D	>0.9999	No	No
FOXP3	autoantibody-positive nT1D vs HC	0.0117	Yes	Yes
IFIT1	T1D vs HC	0.0087	Yes	Yes
IFIT1	T1D vs autoantibody-positive nT1D	0.0045	Yes	Yes
IFIT1	autoantibody-positive nT1D vs HC	>0.9999	No	No
IFI27	T1D vs HC	0.8471	No	No
IFI27	T1D vs autoantibody-positive nT1D	0.0053	Yes	Yes
IFI27	autoantibody-positive nT1D vs HC	0.0003	Yes	Yes
RSAD2	T1D vs HC	>0.9999	No	No
RSAD2	T1D vs autoantibody-positive nT1D	< 0.0001	Yes	Yes
RSAD2	autoantibody-positive nT1D vs HC	0.0002	Yes	Yes
IFI44L	T1D vs HC	>0.9999	No	No
IFI44L	T1D vs autoantibody-positive nT1D	0.0667	No	No
IFI44L	autoantibody-positive nT1D vs HC	0.0795	No	No
ISG15	T1D vs HC	0.0231	Yes	Yes
ISG15	T1D vs autoantibody-positive nT1D	0.8952	No	No
ISG15	autoantibody-positive nT1D vs HC	0.3014	No	No
SIGLEC1	T1D vs HC	0.5286	No	No
SIGLEC1	T1D vs autoantibody-positive nT1D	>0.9999	No	No
SIGLEC1	autoantibody-positive nT1D vs HC	0.2771	No	No

Abbreviations: T1D, established type 1 diabetes; nT1D, new-onset type 1 diabetes; HC, healthy controls.
